# Association between high emotional demand at work, burnout symptoms, and sleep disturbance among Korean workers: a cross-sectional mediation analysis

**DOI:** 10.1038/s41598-023-43451-w

**Published:** 2023-10-04

**Authors:** Seong-Uk Baek, Jin-Ha Yoon, Jong-Uk Won

**Affiliations:** 1 Department of Occupational and Environmental Medicine, Severance Hospital, Yonsei University College of Medicine, 50-1, Yonsei-ro, Seodaemun-gu, Seoul, 03722 Korea; 2 The Institute for Occupational Health, Yonsei University College of Medicine, Seoul, Korea; 3 Graduate School, Yonsei University College of Medicine, Seoul, Korea; 4 Department of Public Health, Graduate School of Yonsei University, Seoul, Korea; 5 Department of Preventive Medicine, Yonsei University College of Medicine, Seoul, Korea

**Keywords:** Epidemiology, Sleep disorders

## Abstract

We explored the mediating role of burnout on the association between EDW and sleep disturbances. Our study included 18,744 Korean workers. Respondents were grouped into four levels (none, low, moderate, and high) based on their exposure to two EDW factors: handling angry clients (HAC) and hiding emotion (HE). Mediation models were used to estimate odds ratios (OR) at a 95% confidence interval (CI). Compared to those not exposed, low, moderate, and high HAC increased odds of sleep disturbance by 1.52 (95% CI 1.30–1.78), 3.20 (95% CI 2.57–3.98), and 3.28 (95% CI 2.77–3.88) times, respectively. The indirect effect via burnout accounted for 29.9%, 24.1%, and 23.6% of the total effect, respectively, with estimates of 1.13 (95% CI 1.11–1.16), 1.32 (95% CI 1.26–1.39), and 1.32 (95% CI 1.27–1.37). Low, moderate, and high HE increased the odds of sleep disturbance by 1.45 (95% CI 0.93–2.28), 2.38 (95% CI 1.57–3.61), and 3.14 (95% CI 2.08–4.71) times, respectively. The indirect effect via burnout accounted for 10.5%, 29.7%, and 33.1% of the total effect, with estimates of 1.04 (95% CI 0.98–1.10), 1.29 (95% CI 1.22–1.37), and 1.46 (95% CI 1.38–1.55), respectively. Effective policies are required to protect the psychological wellbeing of workers who frequently engage in interpersonal tasks.

## Introduction

Emotional demand at work (EDW) refers to the emotional effort required for employees to manage their customers or clients and the negative emotions and worries induced by work^1^. In the realm of protecting workers' mental health, EDW has become a pressing public health concern^2^. According to the International Labour Organization and Eurofound, EDW can be conceptualized based on three aspects: the extent to which workers are required to (1) handle emotionally charged people, (2) suppress their own emotions, and (3) influence the emotions of others^2^. Workers in the healthcare, educational, and service sectors who frequently interact with patients, students, and customers in their jobs often experience high levels of EDW^3^.

Empirical research has demonstrated that EDW exposure is significantly associated with various negative health outcomes. Specifically, individuals exposed to high levels of EDW have a 1.55-fold increased risk of taking long-term sick leave^4^. Moreover, high levels of EDW have been associated with increased risks of hypertension, cardiovascular disease, and musculoskeletal problems^5^. A robust link has been observed between EDW and mental health problems. For instance, high EDW is a well-documented risk factor for depressive symptoms^6^ and suicidal ideation^7–9^. Some studies suggest the association of EDW with an increased risk of sleep disturbances^10,11^. For instance, a previous Korean study revealed that individuals who dealt with angry clients had a higher risk of experiencing sleep disturbance^12^. However, the exact mechanism through which high levels of EDW increase the risk of sleep disturbance is still unknown.

Studies have also shown that EDW is a major risk factor for burnout, which is characterized as a state of emotional and physical exhaustion induced by prolonged exposure to stress^13^. According to previous studies, high EDW, such as dealing with conflicts and tension with clients, can be a contributing factor to burnout^14,15^. Previous studies have indicated that there is a strong correlation between experiencing aggressive customer behavior and the development of symptoms associated with burnout^16,17^.

The literature has demonstrated that prolonged burnout among workers can lead to job dissatisfaction, sickness absenteeism, and mental health problems, including sleep disturbance^18^. Studies have further revealed that burnout plays a mediating role in the relationship between EDW and its adverse effects on mental health. For instance, previous studies have found that burnout partially or fully mediates the association between EDW and negative outcomes, such as turnover intention^19^, poor psychological well-being^20^, and depression^21^. Studies have also found that high burnout symptoms cause sleep disturbances in workers^22,23^. Prolonged burnout symptoms, such as exhaustion, reduce workers’ capacity to cope with stressful situations, thereby intensifying their stress response^24^. Consequently, individuals experiencing burnout may encounter difficulties when trying to initiate sleep and become more susceptible to disruptions in sleep continuity^23,25,26^. However, to the best of our knowledge, the mediating role of burnout in the relationship between EDW and sleep disturbance has been largely unexplored.

This study aimed to examine the association between EDW and sleep disturbance and the potential mediating role of burnout in this relationship. We hypothesized that (1) high EDW is associated with an increased risk of sleep disturbance, and (2) burnout mediates the association between EDW and sleep disturbance. Our study aims to provide policymakers with useful information to strategies to develop effective strategies that can protect workers from sleep disturbances and improve their overall well-being.

## Methods

### Study sample

This study used a sample from the fifth Korean Working Conditions Survey (KWCS), which was conducted in 2017. Since 2006, the KWCS has been conducted every 3 years by the Occupational Safety and Health Research Institute (OSHRI). Benchmarking the European Working Conditions Survey (EWCS), the KWCS aims to gain information about the socioeconomic status, work conditions, and health of workers in South Korea. The target population for this survey comprise workers aged 15 years or older residing in South Korea. The OSHRI employs probability proportional to size sampling method to systemically select a nationally representative sample of workers. Enumeration districts were used as preliminary sampling units, whereas households and household members in each region were selected as secondary sampling units. For selected households, surveys were conducted through face-to-face interviews by trained OSHRI-employed interviewers. The content of the KWCS questionnaire was adapted from the EWCS and subsequently translated into Korean. The KWCS questionnaire was designed to demonstrate high content validity and reliability^27^.

A total of 50,205 participants was initially included in the fifth KWCS. We have included only employed workers (wageworkers) and excluded self-employed workers and unpaid family workers, leaving 30,300 workers. Then, we have excluded blue-collar workers (craft and related trades workers; plant, machine operators and assemblers; and elementary workers), because they may not align with the conventional definition of emotional labor (n = 10,201). After excluding those with missing values (n = 1355), the final sample consisted of 18,744 workers.

### Variables

#### Main variables

This study utilized a cross-sectional mediation analysis, wherein the independent, mediator, and outcome variables were simultaneously assessed during data collection. The Minimal Insomnia Symptom Scale (MISS), was used to assess sleep disturbance, a reliable and widely used tool in epidemiological studies for measuring sleep disruption^28^. The MISS consists of three items that assess the respondents’ sleep disturbance with respect to “difficulty initiating sleep”, “difficulty maintaining sleep” , and “not feeling refreshed after sleep”. Each item was rated on a scale of 0 (“Never”) to 4 (“Every day”), resulting in a total score range of 0–12. Based on previous research^28^, we considered participants with a total score of 6 or higher to have sleep disturbance.

Based on the conceptualization of International Labour Organization and Eurofound^2^, we evaluated the degree of workers’ exposure to EDW by assessing their level of involvement in handling angry clients (HAC) or hiding emotion (HE). First, participants in the KWCS were asked to answer the following question: “Does your job involve handling angry clients, customers, patients, pupils etc.?” Response scale consists of seven options: never, almost never, 1/4 of the work time, 1/2 of the work time, 3/4 of the work time, almost all the time, and all the time. Based on their responses, HAC was categorized into one of four groups: none (never, almost never), low (1/4 of the work time), moderate (1/2 of the work time), and high (3/4 of the work time, almost all the time, all the time). Second, HE was measured from the respondents’ level of agreement with the following statement: “My job requires me to hide my emotions”. HE was measured using a response scale consisting of five options: always, most of the time, sometimes, rarely, and never. Based on their responses, HE was categorized into one of four groups: none (never), low (rarely), moderate (sometimes), and high (most of the times, always). These two items (HAC and HE) have been widely employed in previous Korean studies to measure the health effect of EDW^6,29–31^.

The assessment of burnout was conducted by employing two items derived from the Korean version of the Maslach Burnout Inventory-General Survey^32^. Respondents were asked, “In your job, how often do you feel the following? (1) I feel exhausted at the end of the working day (exhaustion); (2) I doubt the importance of my work (disengagement)”. A previous study confirmed the reliability and validity of this burnout questionnaire^33^. Responses were recorded on a 5-point Likert scale (1: “Never” to 5: “Always”), where a higher score indicated a higher level of burnout. The total sum of the scores, which ranged from 2 to 10, was included as a continuous variable. This measurement has been used in the previous Korean studies to assess burnout symptoms of workers^33,34^.

Survey participants were asked about their sleep disturbances in relation to symptoms within the past 12 months, whereas questions about the EDW factors or burnout were asked regarding their current state, without specifying a particular time frame.

#### Confounders

The selection of confounders was informed by previous studies^6,29–31^, focusing on socio-demographic and occupational characteristics that have the potential to influence our main variables and introduce bias to the estimation, as depicted in the Directed Acyclic Graph (Figure S2). The following confounders were adjusted: sex (male or female), age (< 40, 40–49, 50–59, ≥ 60), education level (having completed middle school or below, high school, or college or above), income (categorized into four groups according to the quartile values of the monthly income, Q1–Q4), marital status (married, unmarried, or others), weekly working hours (≤ 40 h, > 40 h), occupation type (service or sales workers, white collar), and shift work (yes, no).

### Statistical analysis

#### Descriptive analysis

For the descriptive analysis, we first examined the general characteristics of the study sample according to the level of exposure to each EDW factor (HAC or HE). The Chi-square test was used to compare the features of the groups divided by each EDW factor. We then calculated and visualized the mean values of burnout score and prevalence of sleep disturbance according to EDW.

#### Regression analysis

**Figure 1:**
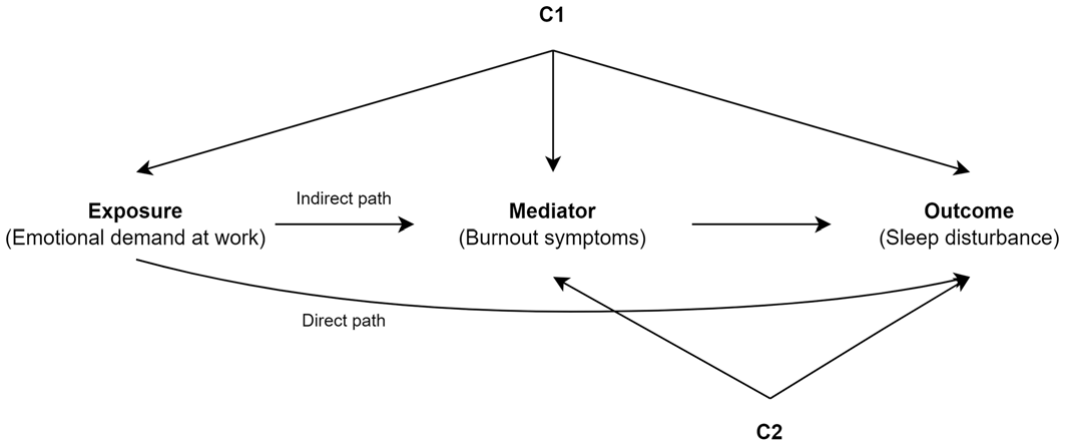
Assumed causal relationship between emotional demands at work (exposure), and sleep disturbance (outcome), mediated through burnout symptoms (mediator). The observed confounder C1 includes sex, age, educational attainment, income level, and marital status, and observed confounder C2 includes working hour, shift work, occupation.

A diagram of the mediation model is shown in Fig. 1. In the preliminary analysis, we examined the significance of associations between variables in the two indirect paths (HAC or HE → Burnout and Burnout → sleep disturbance), by fitting linear and logistic regression models. Specifically, we estimated (1) the association between HAC and burnout, (2) the association between HE and burnout, and (3) the association between burnout and sleep disturbances.

For the main analyses, we fitted logistic mediation models to decompose the total effect into direct (EDW → sleep disturbance) and indirect effects (EDW → burnout → sleep disturbance). Two separate logistic mediation models were fitted to account for the association between HAC or HE and the risk of sleep disturbance. The Stata package “*ldecomp*” was used to perform a simple counterfactual-based logistic mediation analysis using a method proposed by Buis^35^. The effect sizes were presented as odds ratios (OR) with 95% confidence intervals (CIs) of indirect effects estimated through 1000 bootstrap resampling. To calculate the proportion mediated, we divided the indirect effect by the total effect.

$$\mathrm{OR }\left(\mathrm{Total\, effect}\right)=\mathrm{OR }\left(\mathrm{Direct \,effect}\right) \times \mathrm{ OR }\left(\mathrm{Indirect \,effect}\right)$$

$$\mathrm{Proportion \,mediated}=(\mathrm{Indirect \,effect})/(\mathrm{Total \,effect})$$

In the sensitivity analysis, we employed multiple imputation to handle missing observations under missing-at-random assumption. We generated 20 datasets without missing observation and estimates were combined using Rubin’s rule. Stata (version 18.0; StataCorp LLC, College Station, TX, USA) and R (version 4.2.3; R Foundation for Statistical Computing, Vienna, Austria) were used for statistical analyses. Visualizations were performed using R.

### Ethical approval

The Institutional Review Board of Yonsei Health System approved this study (approval number: 4-2021-1046).

## Results

### Descriptive analysis

The participant selection process is illustrated in Figure S1. Table 1 presents the characteristics of the study participants. Of the 18,744 survey participants, 16.3% (N = 3062) were exposed to low HAC, 4.2% (N = 789) to moderate HAC, and 7.5% (N = 1403) to high HAC. Regarding HE, 13.0% (N = 2440), 38.0% (N = 7122), and 44.3% (N = 8305) were exposed to low, moderate, and high HE, respectively. Individuals exposed to a higher level of HAC were more likely to be women, have low income levels, and work more hours weekly. Additionally, those with higher exposure to HE were more likely to have a high level of education and work more hours weekly.

**Table 1:** Baseline characteristics of study participants in the fifth Korean Working Conditions Survey according to emotional demands at work.

Characteristics	Overall	Handling angry clients				P value*	Hiding emotion				P value*
		None	Low	Moderate	High		None	Low	Moderate	High	
	N = 18,744	N = 13,490	N = 3062	N = 789	N = 1403		N = 877	N = 2440	N = 7122	N = 8305	
Sex											
Male	7289 (38.9)	5581 (41.4)	1013 (33.1)	273 (34.6)	422 (30.1)	< 0.001	335 (38.2)	1041 (42.7)	2902 (40.7)	3011 (36.3)	< 0.001
Female	11,455 (61.1)	7909 (58.6)	2049 (66.9)	516 (65.4)	981 (69.9)		542 (61.8)	1399 (57.3)	4220 (59.3)	5294 (63.7)	
Age group											
< 40	8362 (44.6)	5949 (44.1)	1427 (46.6)	369 (46.8)	617 (44.0)	0.002	381 (43.4)	1056 (43.3)	3135 (44.0)	3790 (45.6)	< 0.001
40–49	5385 (28.7)	3925 (29.1)	850 (27.8)	196 (24.8)	414 (29.5)		218 (24.9)	707 (29.0)	2048 (28.8)	2412 (29.0)	
50–59	3880 (20.7)	2762 (20.5)	641 (20.9)	185 (23.4)	292 (20.8)		195 (22.2)	492 (20.2)	1493 (21.0)	1700 (20.5)	
≥ 60	1117 (6.0)	854 (6.3)	144 (4.7)	39 (4.9)	80 (5.7)		83 (9.5)	185 (7.6)	446 (6.3)	403 (4.9)	
Education											
Middle school or below	756 (4.0)	546 (4.0)	107 (3.5)	37 (4.7)	66 (4.7)	< 0.001	59 (6.7)	124 (5.1)	304 (4.3)	269 (3.2)	< 0.001
High school	5219 (27.8)	3596 (26.7)	961 (31.4)	239 (30.3)	423 (30.1)		244 (27.8)	622 (25.5)	1969 (27.6)	2384 (28.7)	
College or above	12,769 (68.1)	9348 (69.3)	1994 (65.1)	513 (65.0)	914 (65.1)		574 (65.5)	1694 (69.4)	4849 (68.1)	5652 (68.1)	
Monthly income (unit: ₩10,000)											
Q1 (≤ 170)	5227 (27.9)	3622 (26.8)	966 (31.5)	215 (27.2)	424 (30.2)	< 0.001	280 (31.9)	664 (27.2)	2004 (28.1)	2279 (27.4)	0.010
Q2 (171–240)	4797 (25.6)	3369 (25.0)	824 (26.9)	218 (27.6)	386 (27.5)		221 (25.2)	592 (24.3)	1829 (25.7)	2155 (25.9)	
Q3 (241–350)	5602 (29.9)	4075 (30.2)	832 (27.2)	260 (33.0)	435 (31.0)		239 (27.3)	728 (29.8)	2091 (29.4)	2544 (30.6)	
Q4 (> 351)	3118 (16.6)	2424 (18.0)	440 (14.4)	96 (12.2)	158 (11.3)		137 (15.6)	456 (18.7)	1198 (16.8)	1327 (16.0)	
Marital status											
Married	13,832 (73.8)	10,056 (74.5)	2234 (73.0)	581 (73.6)	961 (68.5)	< 0.001	642 (73.2)	1811 (74.2)	5259 (73.8)	6120 (73.7)	0.931
Unmarried or others	4912 (26.2)	3434 (25.5)	828 (27.0)	208 (26.4)	442 (31.5)		235 (26.8)	629 (25.8)	1863 (26.2)	2185 (26.3)	
Occupation											
White collar	11,281 (60.2)	8585 (63.6)	1551 (50.7)	407 (51.6)	738 (52.6)	< 0.001	512 (58.4)	1614 (66.1)	4388 (61.6)	4767 (57.4)	< 0.001
Service and sales worker	7463 (39.8)	4905 (36.4)	1511 (49.3)	382 (48.4)	665 (47.4)		365 (41.6)	826 (33.9)	2734 (38.4)	3538 (42.6)	
Weekly working hours											
≤ 40 h	11,529 (61.5)	8558 (63.4)	1744 (57.0)	427 (54.1)	800 (57.0)	< 0.001	566 (64.5)	1590 (65.2)	4513 (63.4)	4860 (58.5)	< 0.001
> 40 h	7215 (38.5)	4932 (36.6)	1318 (43.0)	362 (45.9)	603 (43.0)		311 (35.5)	850 (34.8)	2609 (36.6)	3445 (41.5)	
Shift work											
Yes	16,855 (89.9)	12,415 (92.0)	2586 (84.5)	683 (86.6)	1171 (83.5)	< 0.001	794 (90.5)	2244 (92.0)	6459 (90.7)	7358 (88.6)	< 0.001
No	1889 (10.1)	1075 (8.0)	476 (15.5)	106 (13.4)	232 (16.5)		83 (9.5)	196 (8.0)	663 (9.3)	947 (11.4)	

*Chi-square test.

**Figure 2:**
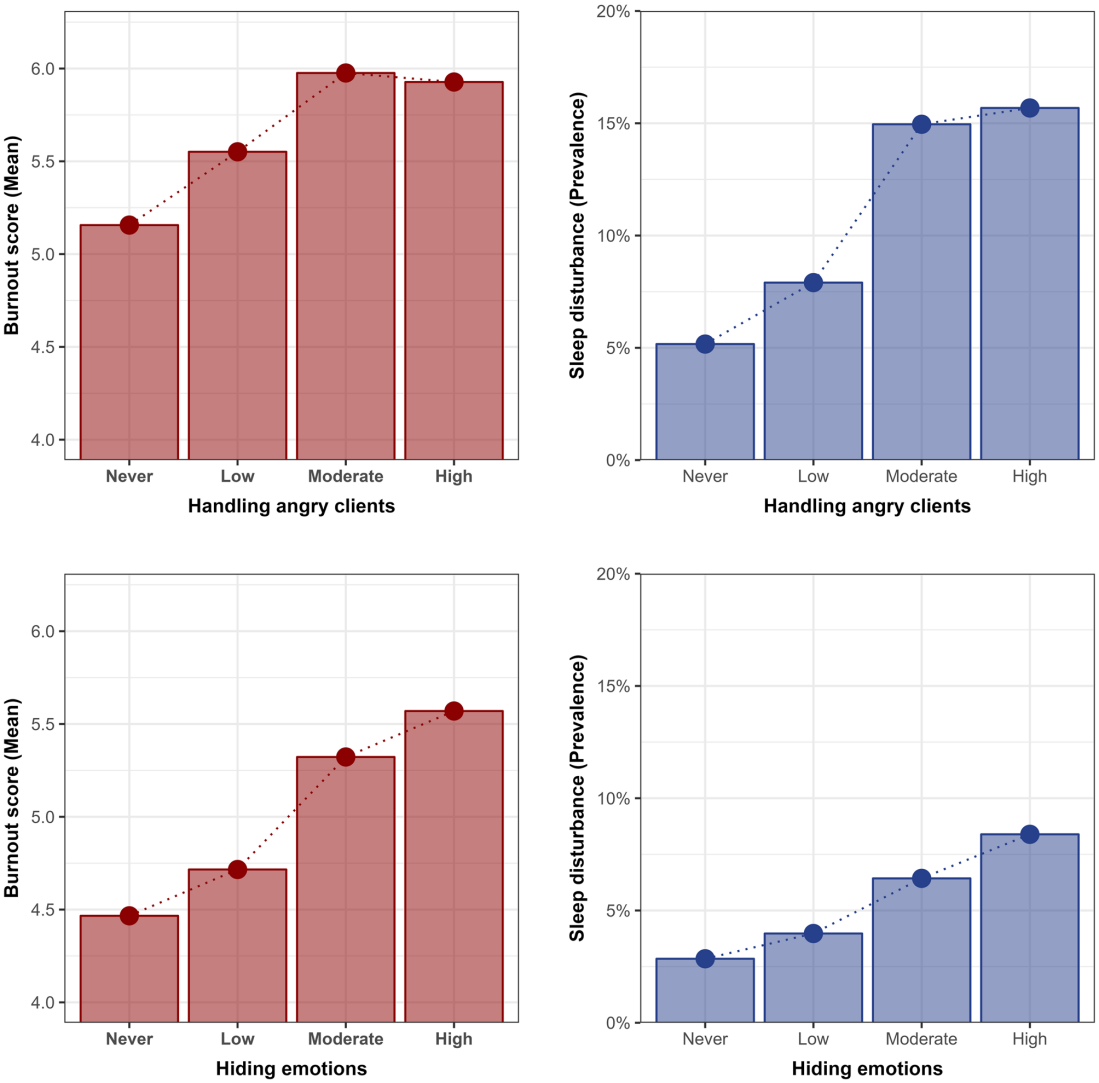
Burnout score and prevalence of sleep disturbance according to emotional demands at work.

Figure 2 shows the mean burnout score and prevalence of sleep disturbance according to the two EDW factors. A dose–response relationship was observed between EDW factors, burnout, and sleep disturbance. For example, those exposed to the highest level of HAC had the high level of burnout (mean = 5.93) and sleep disturbance (prevalence = 15.7%) compared to those not exposed to HAC (burnout: 5.16; sleep disturbance: 5.2%). Similarly, those exposed to the highest level of HE had the high level of burnout (mean = 5.57) and sleep disturbance (prevalence = 8.4%) compared to those who were not exposed to HE (burnout: 4.47; sleep disturbance: 2.9%).

### Preliminary analysis

Table 2 shows the associations between EDW factors with burnout, and burnout with sleep disturbance. In the fully adjusted model, those exposed to low, moderate, and high levels of HAC were associated with a greater level of burnout than those not exposed to HAC (low HAC: *β* [95% CI] = 0.36 [0.29–0.42]; moderate HAC: *β* [95% CI] = 0.78 [0.66–0.89]; high HAC: *β* [95% CI] = 0.73 [0.64–0.82]). Those exposed to low, moderate, and high levels of HE were associated with a greater level of burnout compared to those never exposed to HE (low HE: *β* [95% CI] = 0.26 [0.14–0.38]; moderate HE: *β* [95% CI] = 0.85 [0.74–0.96]; high HE: *β* [95% CI] = 1.08 [0.97–1.19]). Regarding the association between burnout and sleep disturbance, a 1-point increase in burnout score was associated with an elevated OR for sleep disturbance (OR [95% CI]: 1.52 [1.46–1.57]).

**Table 2:** Association of emotional demand at work with burnout and of burnout with sleep disturbance (*EDW* emotional demand at work, *OR* odds ratio, *CI* confidence interval).

Path	Crude model		Adjusted model	
	*β*	95% CI	*β*	95% CI
Model A EDW (handling angry clients) → Burnout				
None	0.00	Reference	0.00	Reference
Low	0.40	0.33–0.46	0.36	0.29–0.42
Moderate	0.82	0.70–0.93	0.78	0.66–0.89
High	0.77	0.68–0.86	0.73	0.64–0.82
Model B EDW (hiding emotion) → Burnout				
None	0.00	Reference	0.00	Reference
Low	0.25	0.13–0.37	0.26	0.14–0.38
Moderate	0.86	0.74–0.97	0.85	0.74–0.96
High	1.10	0.99–1.21	1.08	0.97–1.19

	Crude model		Adjusted model	
	OR	95% CI	OR	95% CI
Model C Burnout → Sleep disturbance				
Burnout (range 2–10)	1.52	1.46–1.58	1.52	1.46–1.57

Model A and B: Linear regression.

Model C: Logistic regression.

The adjusted model controlled for sex, age, education, monthly income, marital status, occupation, working hours, and shift work.

### Mediation analysis

Table 3 shows the total, direct, and indirect effects of each EDW factor on sleep disturbances among workers. A higher level of HAC was associated with an increased risk of sleep disturbance in a dose-dependent manner. For instance, exposure to the highest level of HAC was associated with the greatest OR for sleep disturbance (OR [95% CI]: 3.28 [2.77–3.88]). Compared to those not exposed to HAC, the indirect effect via burnout increased with the level of HAC (low HAC: OR [95% CI] = 1.13 [1.11–1.16]; moderate HAC: OR [95% CI] = 1.32 [1.26–1.39]; high HAC: OR [95% CI] = 1.32 [1.27–1.37]), mediating 29.9%, 24.1%, and 23.6% of the total effect, respectively. A higher level of HE was associated with an increased risk of sleep disturbance in a dose-dependent manner. For instance, exposure to the highest level of HE was associated with the greatest OR for sleep disturbance (OR [95% CI]: 3.14 [2.08–4.71]). Compared to those not exposed to HE, the indirect effect via burnout increased with the level of HE (low HE: OR [95% CI] = 1.04 [0.98–1.10]; moderate HE: OR [95% CI] = 1.29 [1.22–1.37]; high HE: OR [95% CI] = 1.46 [1.38–1.55]), mediating 10.5%, 29.7%, and 33.1% of the total effect, respectively.

**Table 3:** Total, direct, and indirect effect of emotional demand at work on sleep disturbance (*EDW* emotional demands at work, *OR* odds ratio, *CI* confidence interval).

EDW		Total effect		Direct effect		Indirect effect		Proportion mediated
		OR	95% CI	OR	95% CI	OR	95% CI	%
Handling angry clients								
None		1.00	Reference	1.00	Reference	1.00	Reference	
Low		1.52	1.30–1.78	1.34	1.15–1.57	1.13	1.11–1.16	29.9
Moderate		3.20	2.57–3.98	2.42	1.95–3.00	1.32	1.26–1.39	24.1
High		3.28	2.77–3.88	2.48	2.10–2.92	1.32	1.27–1.37	23.6
Hiding emotion								
None	1.00		Reference	1.00	Reference	1.00	Reference	
Low	1.45		0.93–2.28	1.40	0.89–2.19	1.04	0.98–1.10	10.5
Moderate	2.38		1.57–3.61	1.84	1.22–2.79	1.29	1.22–1.37	29.7
High	3.14		2.08–4.71	2.15	1.43–3.23	1.46	1.38–1.55	33.1

Total, direct, and indirect, and total effects were estimated using the counterfactual-based mediation analysis, using “*ldecomp*” package in Stata. The indirect effect quantifies the effect of each EDW factor on the sleep disturbance that is mediated by burnout symptoms.

All model controlled for sex, age, education, monthly income, marital status, occupation, working hours, and shift work.

In the sensitivity analysis (Table S1), the indirect effect of HAC or HE via burnout increased with the level of EDW exposure, accounting for 24.2–32.1% (HAC) and 8.7–32.7% (HE) of the total effects, supporting the findings of the main analyses.

## Discussion

Our study confirmed the mediating role of burnout in the relationship between EDW and sleep disturbance among workers. Our findings indicate a dose–response relationship between EDW exposure and burnout symptoms, as well as the risk of sleep disturbance. Both total and indirect effects increased with higher levels of EDW exposure. This study makes a significant contribution to the current literature by proposing the mediating effect of burnout on the association between EDW and sleep disturbance for the first time.

Our study findings are consistent with previous research showing a significant association between EDW and poor mental health outcomes. Several previous studies have demonstrated that individuals with high EDW are at a greater risk of experiencing depression^6,9,36^ or sleep disturbances^10,37^. Specifically among night shift workers^37^ and customer service workers^10^ in South Korea, high EDW was seen to be associated with sleep disturbance. Similar findings have been reported across studies conducted in different regions, such as the association between high EDW and sleep disturbance in French workers^11^ and Brazilian nurses^38^. Exposure to high EDW can increase work-related stress in workers, which is a well-documented trigger for sleep disturbance^39^.

Our analyses also support the findings of studies that demonstrated positive associations between exposure to high EDW with burnout symptoms. Particularly, our finding that EDW is related to burnout is consistent with earlier studies demonstrating that facing customer aggression can lead to burnout^16,17^. Andel et al.^40^ also found that exposure to emotionally disturbing work is associated with poor sleep quality and high levels of exhaustion. Frequent exposure to EDW requires individuals to invest more emotional, cognitive, or physical energy into coping with tense situations, which can lead to decreased self-work and self-efficacy, resulting in exhaustion and disengagement^14,15,41^.

The relationship between EDW and sleep disturbance mediated by burnout can be elucidated through the Job Demand-Resource (JDR) model, which has been extensively employed to explain the psychological impact of workplace stressors on mental health. According to the JDR theory, an excessive amount of EDW demands emotional, cognitive, and physical energy from workers to resolve conflict, ultimately resulting in exhaustion, which is a key component of burnout^15,42^. In turn, exhaustion makes individuals more vulnerable to stressful events and intensifies their stress response, which eventually leads to difficulties in sleep initiation and high sleep reactivity^23,25,26^. More specifically, workers exhibiting high levels of burnout symptoms may experience negative emotions from their workplace spilling over into their personal lives, causing them to ruminate on stressful events, which contributes to difficulty in initiating or maintaining deep sleep^43^. Similarly, excessive emotional demands can lead to work disengagement, which acts as a work-related stressor causing poor sleep quality^44^.

Our study had several limitations. First, the measurement of EDW utilized in our study were based on single-item indicators. Thus, our current measurement approach may not encompass the entire breadth of the EDW concept. Due to the limitations imposed by the items included in the KWCS, we were unable to employ a multi-item questionnaire with established psychometric properties to comprehensively measure EDW. Consequently, following studies should consider adopting validated instruments capturing the multidimensional nature of EDW, including information about the intensity, patterns, and as well as frequency of EDW exposures. Second, owing to the cross-sectional nature of our analysis, our results do not necessarily represent the causal effects between the variables. The main findings of our study were based on a cross-sectional mediation analysis, wherein the independent, mediator, and outcome variables were simultaneously assessed during data collection. We lack precise information about the specific timing of participants’ exposure to the independent or mediator variables. Consequently, this analysis does not address temporal sequencing and precludes us from making causal assertions about the relationships between these variables. Therefore, further longitudinal studies should be conducted to elucidate temporal dynamics and potential causal mechanisms. Third, there is the possibility of the effects of unmeasured confounders. In particular, previous studies have suggested that individuals with certain personality traits, namely, Type A personality, exhibit high risks of both burnout and sleep disturbance^14,15^. However, our analyses did not consider personality traits owing to lack of available information. Fourth, the measurements used in this study were self-reported by workers; therefore, they were subject to measurement errors and reporting biases. Fifth, as our findings were based on cross-sectional analysis, there is a possibility of reverse causation between sleep disturbance and burnout. Sixth, there is a possibility of biased estimation caused by missing values. Although the sensitivity analysis using multiple imputation confirmed the findings of our main analysis, we could not rule out the possibility of the violation of the missing-at-random assumption, which can cause the biased estimations. Seventh, since the mediation analysis method employed in this study does not perform bootstrapping while considering sampling weight. We could not consider sampling weights in the regression analysis, which may introduce bias to the estimations.

Despite these limitations, our study had several notable strengths. First, our results were derived from a large-scale worker sample representative of the general working population in South Korea. Therefore, the conclusions of the present study have strengths in terms of generalizability. Second, the assessment of sleep disturbance was conducted utilizing a validated instrument. Third, our study is meaningful in that it suggests, for the first time, the mediating role of burnout in the association between EDW and sleep disturbance.

## Conclusion

Our study found that burnout symptoms partially mediated the association between EDW and sleep disturbance. The results suggest that although high levels of EDW may directly contribute to sleep disturbance, they may also indirectly influence it by increasing exhaustion and disengagement among workers. Therefore, policymakers should prioritize the development of strategies and interventions to reduce excess EDW for employees engaged in emotional labor. Furthermore, our results suggest that implementing policy measures to prevent EDW from causing burnout symptoms, such as enhancing organizational support, could be an effective way to reduce the risk of sleep disturbances among employees.

### Supplementary Information

Supplementary Information.

## Data Availability

The raw KWCS data are available online (https://www.kosha.or.kr/eoshri/resources/KWCSDownload.do).
