# Supplementary material for: Association between high emotional demand at work, burnout symptoms, and sleep disturbance among Korean workers: a cross-sectional mediation analysis
Source: Sci Rep. 2023 Oct 4;13:16688. doi: 10.1038/s41598-023-43451-w (PMC10550909; doi:10.1038/s41598-023-43451-w)
Supplement: Supplementary file 1 — Supplementary Information. [file 41598_2023_43451_MOESM1_ESM.docx]

**Association between high emotional demand at work, burnout symptoms, and sleep disturbance among Korean workers: A cross-sectional mediation analysis**

*Supplementary Materials*


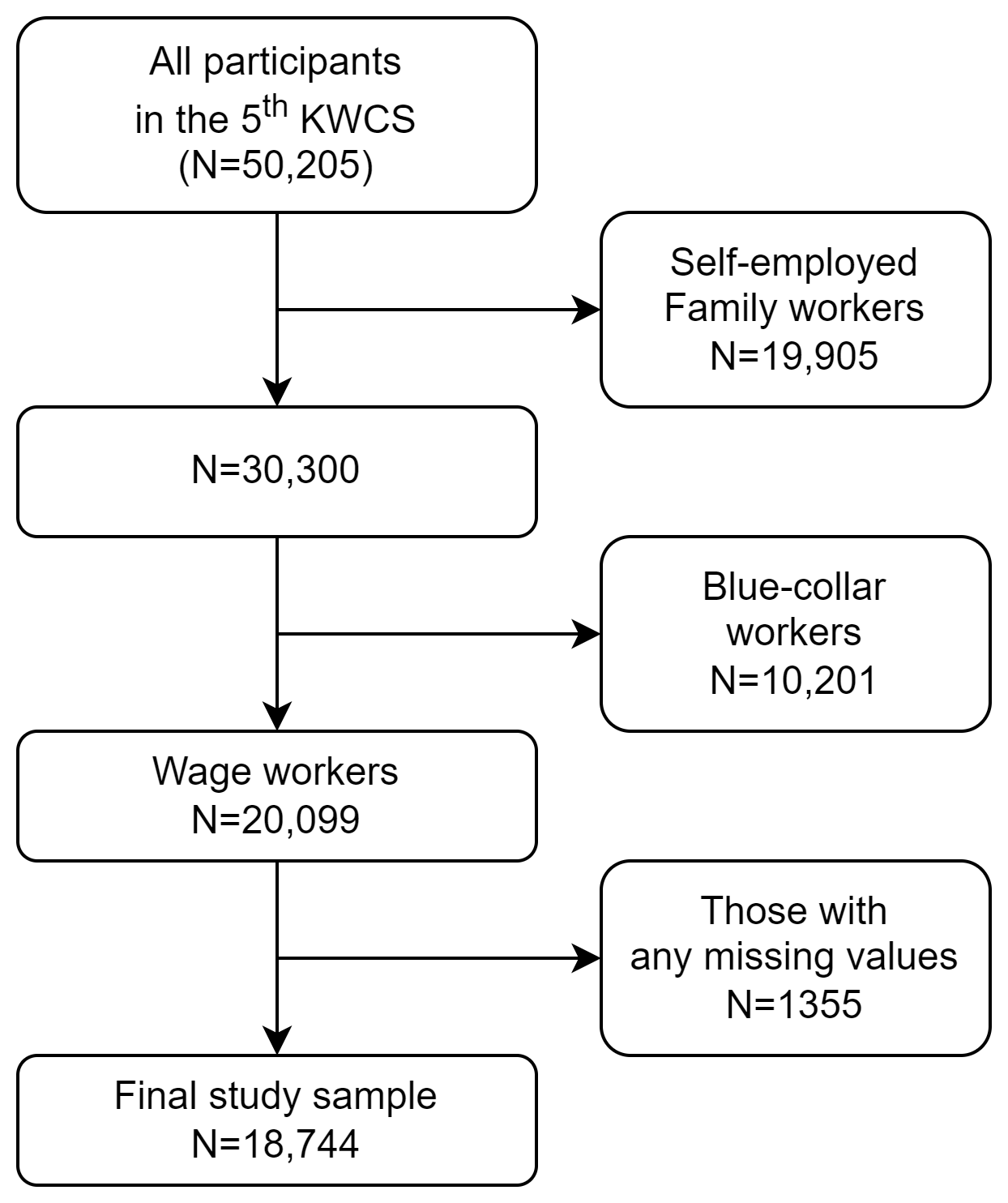


**Figure S1** Flowchart of selection of study sample


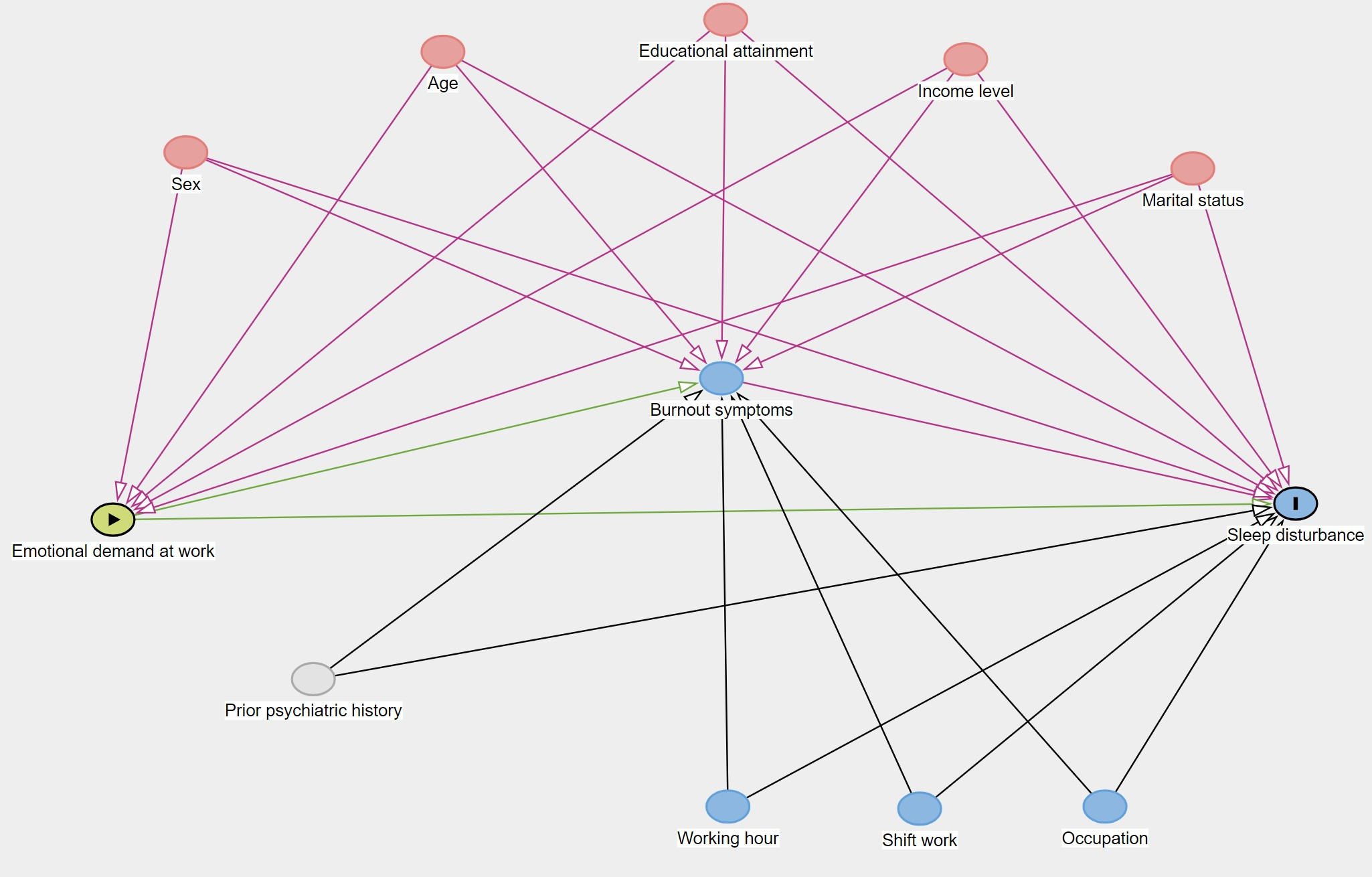


**Figure S2.** Directed acyclic graph of the association between emotional demand at work, burnout symptoms, and sleep disturbance. The variables indicated by translucent gray circles represent potential unobserved confounders (prior psychiatric history).

**Table S1** Results of the sensitivity analysis based on multiple imputation (N: 20,099; OR: odds ratio; CI: confidence interval).

|  | **Total effect** | |  | **Direct effect** | |  | **Indirect effect** | |  | **Proportion mediated** |
| --- | --- | --- | --- | --- | --- | --- | --- | --- | --- | --- |
|  | **OR** | **95% CI** |  | **OR** | **95% CI** |  | **OR** | **95% CI** |  | **%** |
| **Handling angry clients** |  |  |  |  |  |  |  |  |  |  |
| None | 1.00 | reference |  | 1.00 | reference |  | 1.00 | reference |  |  |
| Low | 1.45 | 1.26–1.69 |  | 1.28 | 1.11–1.49 |  | 1.13 | 1.10–1.15 |  | 32.1 |
| Moderate | 3.13 | 2.55–3.83 |  | 2.37 | 1.94–2.90 |  | 1.32 | 1.26–1.38 |  | 24.3 |
| High | 3.14 | 2.68–3.68 |  | 2.38 | 2.04–2.78 |  | 1.32 | 1.27–1.37 |  | 24.2 |
| **Hiding emotions** | | |  |  |  |  |  |  |  |  |
| None | 1.00 | reference |  | 1.00 | reference |  | 1.00 | reference |  |  |
| Low | 1.55 | 1.00–2.41 |  | 1.50 | 0.97–2.31 |  | 1.04 | 0.98–1.10 |  | 8.7 |
| Moderate | 2.52 | 1.68–3.79 |  | 1.94 | 1.29–2.91 |  | 1.30 | 1.23–1.38 |  | 28.5 |
| High | 3.27 | 2.19–4.89 |  | 2.22 | 1.49–3.32 |  | 1.47 | 1.39–1.56 |  | 32.7 |

The adjusted model controlled for sex, age, education, monthly income, marital status, occupation, working hours, and shift work.
